# Effects of Low Molecular Weight Duck Blood Protein Hydrolysate as a Feed Additive on the Intestinal Microbiome, Antioxidant Activity, and Humoral Immune and Inflammatory Responses in Flowerhorn Fish

**DOI:** 10.1155/anu/9970984

**Published:** 2025-04-17

**Authors:** Pimpisut Manassila, Papungkorn Sangsawad, Surintorn Boonanuntanasarn, Jirawadee Kaewda, Pakpoom Boonchuen, Sirawich Limkul, Chatsirin Nakharuthai

**Affiliations:** ^1^School of Animal Technology and Innovation, Institute of Agricultural Technology, Suranaree University of Technology, Muang, Nakhon Ratchasima, Thailand; ^2^School of Biotechnology, Institute of Agricultural Technology, Suranaree University of Technology, Muang, Nakhon Ratchasima, Thailand

**Keywords:** antioxidant, duck blood, immune response, microbiome, protein hydrolysate

## Abstract

Food-derived bioactive peptides could serve as feed ingredients and/or feed additives. We investigated the health-promoting properties of low molecular weight duck blood protein hydrolysate (DBPH), fractionated by ultrafiltration with a 10 kDa molecular weight cut-off membrane, in flowerhorn fish. The analysis of molecular weight distribution revealed that the most common sizes of DBPH fell within the range of 3–7 kDa (39.68%), followed by >7–10 kDa (20.69%), 1–3 kDa (23.03%), and <1 kDa (9.00%). After 1 month of the feeding trial, fish fed with diets supplemented with 2% DBPH exhibited the highest growth, antioxidant activity, and humoral immune response enhancement under normal conditions. In addition, microbiome analysis confirmed that 2% DBPH possesses antimicrobial activity, as evidenced by the significant decrease in operational taxonomic units (OTUs) and alpha diversity indexes, including Chao1 and Shannon. Compared to the control group, fish that were fed with diets supplemented with 2% DBPH exhibited a significantly higher abundance of the genera Cetobacterium and Romboutsia, which could serve as indicators of the overall health and well-being of the fish. After a *Streptococcus agalactiae* challenge, fish fed with diets supplemented with 2% DBPH exhibited an enhanced ability to modulate inflammatory genes, including interleukin (IL)-1*β*, IL-6, CC, and CXC chemokine as well as antioxidant gene expression (superoxide dismutase (SOD) and catalase (CAT)). Overall, dietary supplementation with 2% DBPH could improve the overall health of the flowerhorn fish by ameliorating humoral immune response, alleviating oxidative stress, and strengthening resistance against *S. agalactiae*.

## 1. Introduction

Recently, the price of feed ingredients has begun an unprecedented escalation, leading to an unprecedented rise in feed costs. Using alternative feed materials, particularly food and feed by-products, would enable the reduction of feed costs. Food-derived bioactive peptides could provide feed ingredients and/or feed additives. In Thailand, the duck meat production yield was approximately 74,700 metric tons in 2022 [1], with duck blood being one of the main byproducts. Duck blood contains a richness of essential amino acids, heme iron, and other macro- and micronutrients and can be utilized as an inexpensive protein source with a high percentage of protein when compared to chicken and bovine sources [2]. For these reasons, duck blood byproduct is considered a beneficial protein source whose value increases through hydrolysis as a feed additive in animal diets.

Protein hydrolysates derived from animal byproducts have been reported to be a feasible alternative source of high-quality protein in the diets of the livestock and aquaculture industry sectors [3, 4]. In addition to providing essential nutrient sources and growth factors, protein hydrolysate is also recognized as a value-added product due to its functionalities. The hydrolysis process breaks down proteins into peptides of varying sizes, making them easier to absorb compared to native proteins. Peptides are indeed common products of protein digestion that can enter enterocytes through the peptide transport system according to metabolic physiology. In addition, some peptides also generate a bioactive activity by stimulating the gastrointestinal tract and the immune system to further exert a broad spectrum of functions, including immunomodulatory, antioxidant, anti-inflammatory, and antimicrobial properties depending on their sequence and amino acid composition [5, 6]. Typically, bioactive peptides refer to low molecular weight peptides ranging in size from 2 to 20 amino acid residues, although they can sometimes be larger. In general, it is commonly reported in the literature that potent bioactive peptides typically have a molecular weight below 10 kDa. Since protein hydrolysate consists not only of valuable functional ingredients but also possesses health-enhancing properties, it has been well-demonstrated that it boosts productivity and performance, disease resistance, and immune responses of many fish species, such as common carp [7], Japanese sea bass [8], large yellow croaker [9], Japanese flounder [10], barramundi [11], Gilthead sea bream [12], and Nile tilapia [13]. Although the hydrolysis process increases the price of value-added protein hydrolysate products, it still reached the break-even point for the ornamental fish business due to the high market value of the ornamental fish industry.

The global ornamental fish market is valued at approximately USD 15–30 billion each year and is expected to increase continuously [14]. Consequently, demand for feed additives for ornamental fish has increased. Among ornamental fish species, the flowerhorn fish has emerged as one of the most popular aquarium ornamental fish in the world since it first appeared in 1996 [15]. The price of flowerhorn fish is determined by the uniqueness of the type of fish regarding its size, color, attributes, rarity, individual consumer preferences, healthiness, and its demand in different regional markets. Generally, flowerhorn fish are highly territorial and aggressive, especially in confined spaces. They are known to attack other fish, making them difficult to keep in a community tank. Indeed, flowerhorn fish often suffer from disease and the requirement of dietary prophylaxis in flowerhorn fish is necessary. Recently, numerous studies have investigated the effects of protein hydrolysate on the health of fish, as well as its effect on gut microbiota. Some research has reported that protein hydrolysates can interact with the microbiota in the fish gut, thus, enhancing the epithelial barrier and nutrient absorption and exerting antimicrobial activity by promoting the release of mucus in the intestinal tract [5, 16]. Moreover, the administration of protein hydrolysate can improve the development of the immune system, augmenting immunoglobulin (Ig) and cytokines, including tumor necrosis factor *α* (TNF-*α*), interleukin (IL)-1*β*, and IL-10 production in juvenile barramundi *Lates calcarifer* [6].

To date, several scientific studies have demonstrated that antimicrobial and antioxidant activities were found in animal blood protein hydrolysates, such as those from bovine, chicken, and porcine sources [17–21]. Research on low molecular weight duck blood protein hydrolysate (DBPH) and their application in the ornamental aquaculture field is required. The development of duck blood byproducts as a source of protein and bioactive peptides in the ornamental fish industry through the application of protein hydrolysate technology is a promising and reliable strategy for promoting the health and immunity of fish. We hypothesize that dietary supplementation with low molecular weight DBPH will: (1) positively alter the intestinal microbiome composition of flowerhorn fish, promoting beneficial bacterial populations and enhancing gut health; (2) increase antioxidant activity, as evidenced by elevated levels of antioxidant enzymes and reduced oxidative stress markers; (3) enhance the humoral immune response, leading to improved disease resistance; and (4) modulate the inflammatory response following bacterial infection. Therefore, this study aimed to investigate the optimal level of DBPH as a feed additive in a commercially practical diet and its effects on the intestinal microbiome, humoral immune response, and antioxidant activity in flowerhorn fish. In addition, after 1 month of feeding trials, the fish were intraperitoneally injected with *S. agalactiae*, a significant bacterial pathogen affecting various freshwater fish species, particularly under stress conditions. The expression of antioxidant and inflammatory genes was then analyzed to evaluate the immune response's effectiveness in combating the harmful bacterial infection.

## 2. Material and Methods

### 2.1. DBPH Preparation

The production of DBPH was accomplished through a multistep protocol. Initially, 10 kg of duck blood was subjected to thermal treatment at 90°C for 30 min. The thermally treated duck blood was combined with 50 L of deionized (DI) water. Adhering to the methodology adapted from Sangsawad et al. [22], the Neutrase enzyme, exhibiting an activity of 0.8 AU-N/g, a pH of 7.0, and a temperature of 50°C, was added into the mixture at a 5% ratio relative to the substrate. The resulting mixture underwent enzymatic hydrolysis for 4 h in a water bath stirrer operating at 120 rpm. Subsequently, the enzyme activity was terminated by heating the mixture to 95°C for 30 min. The ensuing step involved centrifuging the mixture at 12,500 rpm for 15 min at 4°C. The supernatant was then separated and transferred to an Amicon Ultra-15 Centrifugal Filter (10 kDa, Merck KGaA, Darmstadt, Germany), wherein it was subjected to further centrifugation at 4000 × *g* for 60 min at 4°C. Ultimately, the permeate fraction with a molecular weight below 10 kDa was collected and analyzed for its molecular weight via sodium dodecyl sulfate polyacrylamide gel electrophoresis (SDS-PAGE) and size exclusion chromatography.

### 2.2. Determination of Protein Molecular Weight and Protein Concentration

The molecular weights of whole duck blood (WB) and DBPH proteins were analyzed using SDS-PAGE, with a constant current of 15 mA for 15 min in the 5% stacking gel and a constant voltage of 100 V for 120 min in the 15% resolving gel. Next, the gel was stained with coomassie brilliant blue R-250 and imaged using the ChemiDoc MP Imaging System (Bio-Rad Laboratories, Hercules, CA, USA). The concentration of DBPH was determined using the Lowry protein assay by measuring absorbance at 750 nm.

### 2.3. Molecular Weight Distribution Analysis

Molecular weight analysis is heavily relied upon for understanding peptide compositions, comparing sizes, and estimating hydrolysis levels. The method outlined by Luasiri et al. [23] was followed and size exclusion chromatography was employed to assess molecular weight distribution. A 100 µL sample containing peptides (10 mg/mL) was subjected to chromatography using a Superdex Peptide 10/300 GL column (GE Healthcare, Piscataway, New Jersey, USA) at a 0.7 mL/min flow rate. Isocratic elution was performed with a solution comprising 30% acetonitrile and 0.1% trifluoroacetic acid in DI water, totaling 30 mL. The peptide profile was determined by measuring UV_215_ nm absorption, with cytochrome-c, aprotinin, synthetic peptides, and tyrosine used as the standards for determining molecular weight.

### 2.4. Diet Preparation

The experimental diets were designed for five treatment groups: a commercial diet (negative control), a commercial diet supplemented with 0.1% (w/w) vitamin C (Stay C-35, F. Hoffmann-La Roche, Basel, Switzerland; positive control), and a commercial diet supplemented with three levels of 0.5%, 1%, and 2% DBPH. The concentration of DBPH in each diet preparation was strictly measured using the Lowry protein assay, as described previously. All experimental diets were coated with 2% (v/w) squid oil and air-dried at room temperature for 2 h. After that, the experimental diets were aliquoted for daily use and stored at 4°C until used. The major chemical compositions of each experimental diet group were analyzed according to the standard methods outlined by the Association of Official Analytical Chemists (AOAC; 1990), which comprised 38% crude protein, 10% moisture, 4% crude fiber, and 4% crude fat. No significant difference was observed among experimental diets. All fish in each treatment were fed ad libitum twice daily throughout the experimental period.

### 2.5. Experimental Design, Fish Culture, and Sampling

Before starting the experiment, 40 healthy flowerhorn fish (weighing 3.34 ± 0.67 g and measuring 3.40 ± 0.61 cm in length) were acclimatized in 40 glass aquarium tanks (23″ × 13″ × 12″) at the Suranaree University of Technology Farm in Nakhon Ratchasima Province, Thailand, for 14 days. Since flowerhorn fish have aggressive temperaments and can potentially harm one another, the fish were individually separated in each tank. The experimental animals were assigned to five experimental diet groups, each with eight replicated fish, using a completely randomized design. Throughout the experimental period, the fish were raised in aquarium tanks with temperature control, aeration, and water filtration systems. After 1 month of the feeding trial, weight gain rate (WGR), final body length, and survival rate (SR) were recorded (*n* = 8). The growth parameters were calculated as follows: WGR = final weight (g) − initial weight (g); SR (%) = 100 × final number (fish)/initial number (fish).

After that, the fish in each treatment were split into a prechallenge group (*n* = 4) and a postchallenge group (*n* = 4). In the prechallenge group, serum was collected from the fish in each experimental group for humoral immune responses and antioxidant enzyme activity analysis. The DBPH group that showed the best results in terms of growth, humoral immune responses, and antioxidant enzyme activity (described in Section 3) was used for further analysis as the optimal percentage DBPH group. The intestines were collected from the negative control and the optimal percentage DBPH group for microbiome analysis. The liver and spleen were collected from the negative control, positive control, and the optimal percentage DBPH group for gene expression analysis to compare with the postchallenge group. In the postchallenge group, fish from the negative control group, positive control group, and optimal percentage DBPH group were intraperitoneally injected with 10^8^ CFU/mL of *S. agalactiae* and liver and spleen samples were collected at 24 h postinjection. During all sampling procedures, fish were anesthetized with 2-phenoxyethanol at 0.35 mL/L and euthanized using an overdose of 1 mL/L.

### 2.6. Immune Responses and Antioxidant Enzyme Activity

Blood samples from four fish in each treatment were withdrawn from the caudal vein under anesthesia, left at room temperature for 2 h to separate the serum, and then stored at −80°C until use. The total Ig, lysozyme activity, and alternative complement (ACH50) activity were determined from the serum of flowerhorn fish. Total Ig was measured using the total protein kit (Biuret method; Erba, Mannheim, Germany). Lysozyme activity was measured according to the method of Siwicki and Studnicka [24], while ACH50 activity was determined following a method by Milla et al. [25]. Catalase (CAT), superoxide dismutase (SOD), and malondialdehyde (MDA) levels were quantified in the serum of flowerhorn fish using commercial kits (Abbkine Corporation, GA, USA), according to the manufacturer's recommended protocol.

### 2.7. Intestinal Microbiota Analysis

Intestines from three fish in each group, including those on the optimal DBPH-supplemented diet and the negative control diet, were used for genomic DNA extraction using a FavorPerp Stool DNA Isolation Mini Kit (Farvogen Biotech Corp, Ping Tung, Taiwan) with some modifications. To determine the quantity and quality of genomic DNA, 2 µL of each gDNA sample was analyzed using a NanoDrop 2000 Spectrophotometer (Thermo Fisher Scientific, Waltham, MA, USA) and the integrity of the gDNA was assessed through agarose gel electrophoresis. Subsequently, three DNA libraries from the control group and another three from the DBPH group were amplified using the V3–V4 region of the bacterial 16S ribosomal RNA gene. The libraries were prepared and sequenced on Illumina Novaseq 6000 and data were then analyzed by bioinformatics (Biomarker Technologies BMKGENE; Münster, Germany).

To generate high-quality reads, the raw reads were filtered by trimmomatic v0.33, and the primer sequences were removed by cutadapt 1.9.1. Next, Dada2 in QIIME2 was performed to de-noise and remove chimeric sequences. Taxonomic annotation was conducted on operational taxonomic units (OTUs) using the SILVA reference database. The abundance of each species in the samples was calculated at the phylum, class, order, family, genus, and species levels using statistical analysis based on the composition of each sample. The abundance of data was generated by the R package for the characterization of microbial communities present in the samples. To measure species diversity within individual samples, alpha diversity analysis was assessed using various metrics, including the Chao1, Shannon, and Simpson indices. Conversely, beta diversity analysis was assessed to compare species diversity between different samples using principal component analysis (PCA) generated by the R package.

### 2.8. Challenge Trial

#### 2.8.1. Preparation of *Streptococcus agalactiae*

The *S. agalactiae* virulent strain was isolated from the infected fish from an earthen culture pond in the northeast part of Thailand and maintained in the Laboratory of Biotechnology for Aquaculture, Suranaree University of Technology. A single colony was resuspended in tryptic soy broth (Merck KGaA, Darmstadt, Germany) and cultured at 37°C and 150 rpm for 16–18 h. The bacterial suspension was adjusted to a final concentration of 1 × 10^8^ CFU/mL with an optical density at 600 nm of 1.0.

#### 2.8.2. Inflammatory and Antioxidant Gene Expression

To evaluate the impact of DBPH on mRNA expression levels of inflammatory and antioxidant genes after the feeding trial, liver, and spleen tissues were collected from each experiment (*n* = 4), both pre- and postchallenge. These samples were placed in microcentrifuge tubes with 1 mL of Tri-reagent (Molecular Research Center, Inc., OH, USA) for total RNA extraction, according to the manufacturer's instructions. The total RNA was then synthesized into complementary DNAs (cDNAs) using Viva cDNA Synthesis Kit (Vivantis Technologies, Selangor, Malaysia) to determine the expression levels of inflammatory (IL-1*β*, IL-6, CC, and CXC chemokine) and antioxidant (CAT and SOD) genes using absolute real-time quantitative PCR (qRT-PCR). Subsequently, partial fragments of these genes and the *β*-actin gene were amplified from the liver and spleen cDNA using specific primer sets as illustrated in Table 1. The *β*-actin gene was used as an internal reference gene for normalization. The purified PCR products were cloned into pGEM T-Easy plasmid (Promega Corporation, Madison, WI, USA) and the plasmid clones were sequenced by Macrogen, Inc. (Seoul, Korea) to validate the accuracy of the standard plasmids for qRT-PCR analysis. The CFX Opus Real-Time PCR System (Bio-Rad, Hercules, CA, USA) and THUNDERBIRD SYBR qPCR Master Mix (TOYOBO, Osaka, Japan) were employed to investigate the mRNA expression of antioxidant genes (in triplicate) according to our previous study [26].

### 2.9. Statistical Analysis

SPSS 25.0 software (SPSS Inc., Chicago, IL, USA) was used for statistical analyses. The results are reported as least squares mean values, accompanied by the residual standard error (RSE). The data was analyzed using a one-way analysis of variance, according to the complete randomized design (CRD) experimental plan to compare the differences between the means in each experimental group, using Tukey's multiple tests at the accepted significance level of *p* < 0.05. *In addition*, an independent sample *T*-test was conducted to evaluate the difference between the negative control group and the optimal percentage DBPH group for the microbiome analysis, as well as between the pre-and postchallenge group for gene expression analysis (*p* < 0.05).

## 3. Results

### 3.1. Determining the Molecular Weight of DBPH and Peptide Distributions

**Figure 1:**
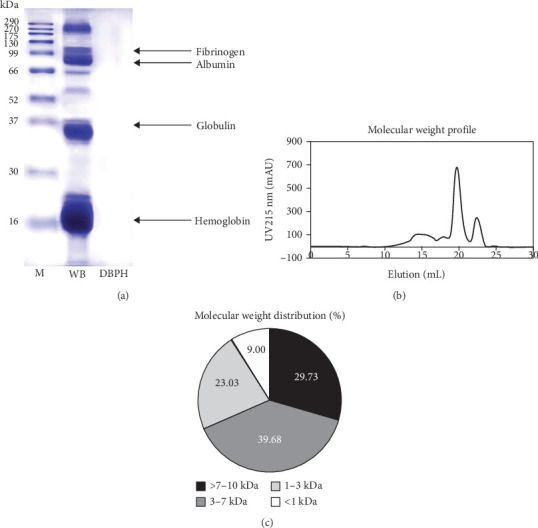
The SDS-PAGE profile (12.5% acrylamide) of samples containing 30 µg of protein (A). The samples include protein standard markers (M), whole duck blood (WB), and low molecular weight duck blood protein hydrolysate (DBPH). Additionally, size exclusion chromatography was used to obtain the molecular weight profile (B) and molecular weight distribution (C) of DBPH peptides.

The protein profiles of WB and DBPH samples were analyzed using SDS-PAGE, as shown in Figure 1A. In the WB lane, prominent proteins, such as hemoglobin monomer, globulin, albumin, and fibrinogen, were observed. In contrast, the DBPH lane exhibited no discernible protein bands, indicating complete protein degradation. In this instance, the peptides had a molecular weight below 10 kDa. The molecular weight profile of peptides obtained from the DBPH sample via size exclusion chromatography is depicted in Figure 1B, while the corresponding calculated molecular weight distribution is presented in Figure 1C. Upon analyzing the distribution of molecular weights, it was evident that the most common sizes fell within the range of 3–7 kDa (39.68%), followed by >7 kDa (20.69%), 1–3 kDa (23.03%), and <1 kDa (9.00%).

### 3.2. Growth and SR

**Figure 2:**
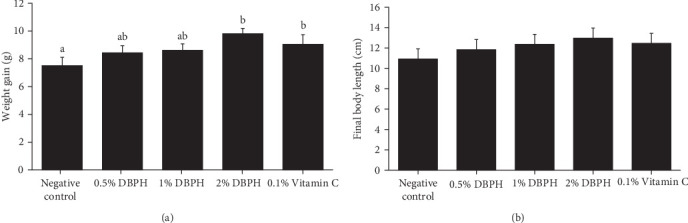
Weight gain (A) and final body length (B) of flowerhorn fed with different experimental diets for 30 days. Means ± S.D. (*n* = 8) with different superscript letters are significantly different (*p* < 0.05).

At the end of the feeding trial, the results showed that weight gain had significantly increased in the fish that were fed diets supplemented with 2% DBPH and 0.1% vitamin C (*p* < 0.05) compared to the control group, while final body length did not differ among the experimental groups (Figure 2). The SR of all experimental groups was 100%.

### 3.3. Immune Responses

Only the group supplemented with 2% DBPH in the diet showed a significant increase in ACH50, lysozyme activity, and total Ig levels compared to the negative control group. In addition, there was no significant difference between the group supplemented with 2% DBPH and the group supplemented with 0.1% vitamin C (positive control), as shown in Table 2.

### 3.4. Antioxidant Activity

Overall, the groups supplemented with 1% and 2% DBPH in the diet exhibited a significant increase in SOD and CAT values, while the MDA value also significantly decreased in the same groups compared to the negative control group. In addition, there was no significant difference between the group supplemented with 1% and 2% DBPH and the group supplemented with 0.1% vitamin C (positive control), as shown in Table 3.

### 3.5. Intestinal Microbiota and Diversity Analysis

A total of 480,439 raw reads were generated from six libraries, comprising three libraries of the 2% DBPH group and another three libraries in the control group. The average raw read, clean read, and tags of each group are displayed in Table 4. The 2% DBPH group possessed significantly fewer unique OTUs than the control group. The alpha diversity indexes, including Chao1 and Shannon, of the microbiota in flowerhorn fed with dietary supplementation of 2% DBPH were significantly lower than those of the control group, while the variation in the Simpson index was not significantly different.

**Figure 3:**
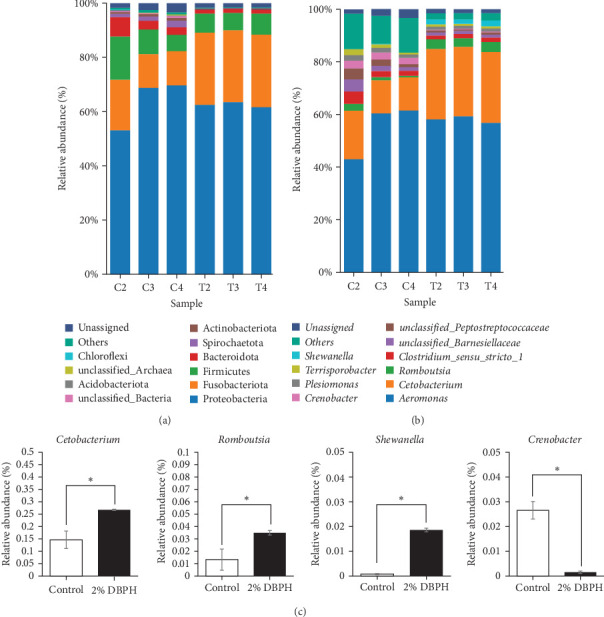
Intestinal microbiota composition of flowerhorn fish fed with the control diet and 2% DBPH for 30 days. Taxonomic distribution at phylum level (A), taxonomic distribution at genus (B), and abundance of significant intestinal bacteria communities at the genus level (C). *⁣*^*∗*^Indicates a statistically significant difference at *p* < 0.05.

**Figure 4:**
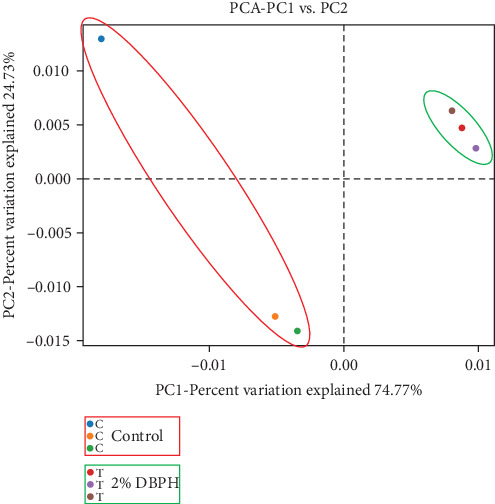
Principal component analysis (PCA) based on distances of intestinal bacteria communities of flowerhorn fish fed with the control diet and 2% DBPH for 30 days.

The relative abundance (%) at the phylum level is shown in Figure 3A. The result demonstrated that proteobacteria (control = 63.84% ± 9.20% and 2% DBPH = 62.47% ± 1.00%), fusobacteriota (control = 14.63% ± 3.55% and 2% DBPH = 26.75% ± 0.26%), firmicutes (control = 10.34% ± 5.01% and 2% DBPH = 7.03% ± 0.54%), bacteroidota (control = 4.36% ± 2.34% and 2% DBPH = 1.42% ± 0.12%), and actinobacteriota (control = 0.78% ± 0.18% and 2% DBPH = 0.22% ± 0.01%) phyla were the most abundant in these experimental groups. Aeromonas, cetobacterium, romboutsia, clostridium_sensu_stricto_1, unclassified_Barnesiellaceae, unclassified_Peptostreptococcaceae, crenobacter, plesiomonas, terrisporobacter, and shewanella were the most plentiful at the genus level (Figure 3B). In addition, four genera, including cetobacterium, romboutsia, crenobacter, and shewanella, showed significant differences between the control group and the 2% DBPH group, as shown in Figure 3C. The beta diversity analysis presented by PCA revealed complete separation in the clustering of intestinal microbiota between the control and 2% DBPH groups (Figure 4).

### 3.6. Antioxidant Gene Expression in Response to *S.agalactiae*

**Figure 5:**
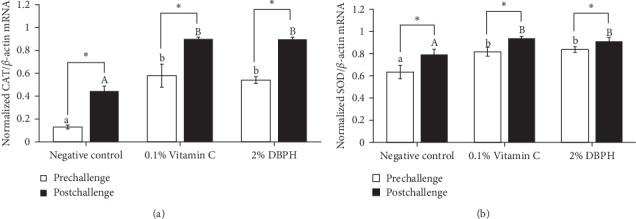
Quantitative real-time PCR analysis of CAT (A) and SOD (B) expression in the liver of flowerhorn fish after the 30-day feeding trial. Bars with asterisks indicate significant differences between pre- and postchallenge. Bars labeled with different lowercase letters denote significant differences at the prechallenge stage, while bars labeled with uppercase letters indicate significant differences at 24 h postchallenge (*p* < 0.05).

Regarding antioxidant gene expression, CAT and SOD mRNA levels in the liver of fish fed with dietary supplementation with 2% DBPH for 30 days (prechallenge) increased in comparison to the negative control group. Moreover, at 24 h postinjection, the expression of CAT and SOD in response to the infection was still significantly higher than in the control group. However, the expression levels of SOD and CAT mRNA in the liver in all groups were significantly increased at 24 h postinjection compared to the baseline (prechallenge; Figure 5A,B).

### 3.7. Inflammatory Gene Expression in Response to *S.agalactiae*

**Figure 6:**
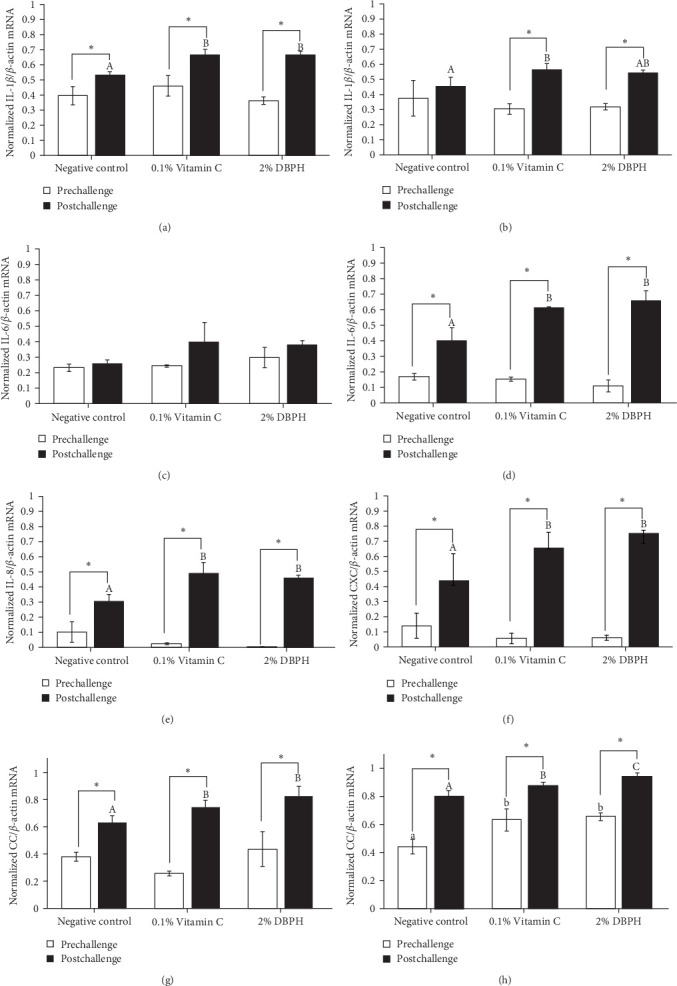
Quantitative real-time PCR analysis of IL-1*β*, IL-6, CC, and CXC chemokine expression in the liver (A, C, E, G) and IL-1*β*, IL-6, CC, and CXC chemokine expression in the spleen (B, D, F, H) of flowerhorn fish after the 30-day feeding trial. Bars with asterisks indicate significant differences between pre- and postchallenge. Bars labeled with different lowercase letters denote significant differences at the pre-challenge stage, while bars labeled with uppercase letters indicate significant differences at 24 h postchallenge (*p* < 0.05).

After 30 days of the feeding trial (prechallenge), the expression of inflammatory genes, including IL-1*β*, IL-6, CC, and CXC chemokine, was investigated (Figure 6). There was no significant difference in the mRNA expression levels in the liver and spleen between the experimental groups except for CC chemokine in the spleen, which showed higher upregulation in 2% DBPH and vitamin C groups compared to the negative control group (Figure 6H). After 24 h postchallenge, IL-1*β*, CXC, and CC chemokine mRNA expression levels increased in both spleen and liver tissues, with greater upregulation in the 2% DBPH and vitamin C groups compared to the negative control group. However, the IL-6 expression level was upregulated only in the spleen (Figure 6D). In comparing individual treatments between pre- and postchallenge, significantly higher expression levels of IL-1*β*, CXC, and CC chemokine were found in both the spleen and liver across all treatments (Figure 6), except for IL-1*β* levels in the negative control group (Figure 6B). However, no significant difference was found in IL-6 levels in all treatments in the liver (Figure 6C).

## 4. Discussion

Protein hydrolysates from animal blood byproducts have gained attention as promising aquaculture feed additives [17–21, 27, 28]. Duck plasma hydrolysate exhibited strong antioxidant properties and a beneficial amino acid composition [29]. However, industrial-scale production faces challenges such as anticoagulant contamination, which increases sodium levels, and the need for strict temperature control during plasma separation, complicating the process and raising costs. This study introduces an innovative approach using WB without anticoagulants. We employed thermal denaturation (90°C for 30 min) to inactivate fibrinogen and reduce microbial load, followed by Neutrase-mediated hydrolysis at pH 7. This process eliminates the need for pH adjustment, avoiding sodium chloride contamination typically associated with acid–base neutralization. Ultrafiltration with a 10 kDa cut-off membrane yielded a low molecular weight of the DBPH, with approximately 32% of peptides below 3 kDa. These small peptides (<3 kDa) demonstrate superior antioxidant activity compared to larger peptides [30], enhancing DPPH performance, metal chelating, and hydroxyl radical scavenging activities [31]. They also exhibit remarkable bioavailability and efficient intestinal absorption, similar to fish protein hydrolysates [32]. Notably, peptides below 1.4 kDa show significant ABTS radical scavenging ability [30]. Given these advantageous characteristics, our low molecular weight DBPH presents a promising bioactive feed additive for aquaculture. Its unique properties and potential benefits warrant further investigation and development for commercial applications.

In the feeding trial, the fish-fed vitamin C supplementation group was used as a positive control. Previous studies indicate that vitamin C and protein hydrolysates exert their effects through similar biological pathways, such as enhancing antioxidant activity and modulating immune responses [29–34]. Therefore, using vitamin C as a positive control aids in clarifying the specific contributions of DBPH in this study. In addition, vitamin C is less stable due to its chemical structure and susceptibility to environmental factors. Consequently, DBPH may represent an alternative source of novel and potent antioxidants. After a 30-day feeding trial, the fish that were fed a diet supplemented with 2% DBPH exhibited superior weight gain and humoral immune response (lysozyme, ACH50, and total Ig) to those fed diets containing 0.5% and 1% DBPH, as did the control groups. Furthermore, adding 2% DBPH in the diet showed no significant difference in the positive control group. In this sense, a higher concentration of low molecular weight bioactive peptides could enhance nutrient absorption, resulting in improved weight gain. Dietary supplementation with 2% DBPH could improve the lysozyme and ACH50 activities, as well as the total Ig levels in the flowerhorn fish compared to the control group. In addition, no significant difference was observed when compared to the group supplemented with vitamin C (positive control). Low molecular weight DBPH could enhance the bioavailability and absorption of nutrients, which are essential for the synthesis of humoral immune protein; consequently, their increased availability contributes to improving the immune function and acts as a defense against pathogens [9, 35]. Additionally, undergoing hydrolysis, DBPH may possess bioactive activities, such as antimicrobial or immunomodulatory effects, which can directly support or enhance the humoral immune response in flowerhorn fish [9, 36]. These findings could confirm the bioactive activity of DBPH.

Regarding antioxidant activity, our previous study [37] identified antioxidant peptides in DBPH, including WMHVR, YAHVR, MPFKY, PDDPR, and NKVHF. These peptides exhibited strong ABTS radical scavenging activity with IC50 values ranging from 0.47 to 5.82 mg/mL, which can reduce oxidative stress-related damage by scavenging free radicals. In our present study, dietary supplementation with 1% and 2% DBPH could enhance the ability of antioxidant enzymes including CAT, SOD, and MDA, with no significant difference observed compared to the group supplemented with powerful antioxidant vitamin C. CAT and SOD function as antioxidant enzymes that aid in neutralizing reactive oxygen species (ROS) within cells, while MDA is a marker of lipid peroxidation, indicative of oxidative stress. These enzymes play a crucial role in the antioxidant defense system of cells. Bioactive peptides derived from hydrolysis can directly scavenge ROS within cells and regulate gene expression related to antioxidant enzymes, leading to increased production and activity of CAT and SOD. In addition, DBPH may possess the ability to inhibit lipid peroxidation, thereby, reducing the formation of MDA. Overall, humoral immune response and antioxidant activity results demonstrated that 2% DBPH is the optimal level of supplementation as a bioactive feed additive for enhancing both antioxidant activity and humoral immune response in flowerhorn fish under these experimental conditions.

Similar to livestock animals, dietary nutrient composition in aquafeed is one of the most important criteria in shaping the composition and function of the gut microbiota in fish, which in turn influences the immune system. To further improve the overall health of fish, the interaction between gut microbiota and fish immunity has garnered interest from nutritional researchers. The intestine provides a living environment for a diverse community of microorganisms, known as the gut microbiota, as well as a large number of immune cells that defend against harmful substances. Evidence has been reported that the optimal amount of protein hydrolysate and its bioactive compounds can improve gut microbiota and enhance immune system function [38–41]. In this study, fish fed with 2% DBPH exhibited a reduction in the variety of intestinal microbiota as indicated by OTUs and the alpha diversity analysis, including Chao1 and Shannon. These results correlated with those reported for turbot (*Scophthalmus maximus*) [41] and largemouth bass (*Micropterus salmoides*) [39], suggesting that low molecular weight DBPH could contain the antimicrobial peptides (AMPs). However, further studies are needed to characterize the sequences of peptides in DBPH that possess antimicrobial activity. In this context, AMPs, which play a crucial role in defending against pathogens, could potentially reduce the biodiversity of microorganisms in the intestines of fish fed with 2% DBPH. This reduction may occur through the disruption of cell membranes or interference with essential microbial functions. In the present study, the dominant phyla in both the unsupplemented DBPH group and the group supplemented with 2% DBPH were proteobacteria, fusobacteriota, firmicutes, bacteroidota, and actinobacteriota. These findings were consistent with the previous studies on turbot (*S. maximus*) [41], and largemouth bass (*M. salmoides*) [39, 42]. At the genus level, fish fed with a 2% DBPH-supplemented group exhibited a significantly higher abundance of cetobacterium, romboutsia, and shewanella, but lower levels of crenobacter compared to the control group. Within the fish gut, the genus *Cetobacterium* plays a role in nutritional utilization by enhancing the digestion and metabolism of both carbohydrates and proteins, especially those molecules that are difficult to break down [43]. In addition, it has been well-demonstrated that cetobacterium is a producer of short-chain fatty acids (butyrate), and vitamin B12. Some studies suggest that certain species of cetobacterium may produce antimicrobial compounds that could potentially help modulate the microbial community in the gut environment by inhibiting the colonization and proliferation of pathogenic species [44]. Hence, the abundance of cetobacterium in the fish gut could be an indicator of the overall health and well-being of the fish [43, 45]. The genus *Romboutsia* is capable of breaking down complex carbohydrates and fermenting amino acids in the gut environment [46]. These processes contribute to the metabolic activity of the gut microbiota and may influence various aspects of host health and physiology. The genera *Shewanella* and *Crenobacter* are occasionally found in the gut microbiota of fish, while commonly found in aquatic environments. However, the exact role of these genera in the fish gut microbiota and their impact on host health and physiology are still not fully understood. Their effects depend on several factors, such as diet, environmental conditions, and the species of fish. Some species of Shewanella have been reported as probiotics in fish [47]; however, other species have been associated with fish diseases [45].

To investigate the role of low molecular weight DBPH as an immunomodulator during pathological conditions, the experimental fish were intraperitoneally injected with *S. agalactiae*. In this study, the mRNA expressions of antioxidant genes (SOD and CAT) and inflammatory cytokines (IL-1*β*, IL-6, CC, and CXC chemokine) were determined at the 30-day feeding trial (prechallenge) and 24 h (postchallenge) after the *S. agalactiae* injection. At the prechallenge stage, significantly higher expression levels of SOD and CAT were observed in the 0.1% vitamin C and 2% DBPH-supplemented groups compared to the negative control group. This suggests that both vitamin C and DBPH may promote antioxidant activity and strengthen cellular defenses against oxidative stress.

During bacterial infection, significantly higher upregulation of antioxidant gene expressions was found in all experimental groups (negative control, 0.1% vitamin C, and 2% DBPH). This demonstrated the crucial role of CAT and SOD genes in immune responses to eliminate ROS during bacterial infection. Interestingly, the expression levels of CAT and SOD were significantly higher in the groups supplemented with 0.1% vitamin C and 2% DBPH compared to the negative control group. This could be attributed to the antioxidant properties of DBPH and vitamin C that support the innate immune response of fish.

In innate immunity, cytokines are critical for initiating immune defense by recruiting the immune cells to the infection site and coordinating subsequent immune responses as a link between innate and adaptive immune responses. In this study, at the prechallenge stage, only the expression of the CC chemokine gene was significantly higher in the spleen of fish-fed dietary vitamin C and DBPH supplementation compared to the control group. This could suggest the ability of vitamin C and DBPH to influence CC chemokine production in the spleen of flowerhorn fish. According to current information, the spleen plays a crucial role in modulating homeostatic conditions through the circulation of CC chemokines [48]. Given that the spleen is the predominant lymphatic tissue in teleost fish, it serves as the site for phagocytic, lymphocytic, and dendritic cells, which are sources of CC chemokines. In addition, other inflammatory genes, including IL-1*β*, IL-6, and CXC chemokine, also exhibited constitutive expression patterns in both the liver and spleen, which are lymphoid organs in flowerhorn fish. This finding demonstrates the functionality of these cytokine genes to continuously balance immune responses under normal conditions. The constitutive expression of cytokines involves their steady-state production and is expressed at relatively constant levels without requiring an inducer or stimulus under normal physiological conditions [49, 50]. Similarly, in other fish species, the constitutive expression of these cytokine genes has also been observed primarily in immune organs. This indicates their role in immune surveillance and maintaining tissue homeostasis in flowerhorn fish. At the postchallenge stage, the significantly higher mRNA upregulation levels of IL-1*β*, CC, and CXC chemokine persisted in both the liver and spleen among the vitamin C and DBPH-supplemented groups compared to the control group. In the case of IL-6, a significantly higher expression persisted only in the spleen at 24 h, indicating that this organ plays a vital role in the regulation of IL-6 expression in response to *S. agalactiae* in flowerhorn fish. As part of the innate immune response, the upregulation of cytokines occurs rapidly, ranging from hours to days following bacterial injection, to recruit the immune cells to the infection site. In this context, DBPH may enhance pro-inflammatory gene expression in response to pathogenic bacteria in this study. Similarly, the supplementation of other protein hydrolysates at suitable levels has been convincingly demonstrated to enhance disease resistance in Japanese sea bass [8], turbot [41], European sea bass [40], red sea bream [38], and barramundi [51]. Furthermore, a significant increase in the mRNA expression level between pre- and postchallenge was observed for CC and CXC chemokine in both the spleen and liver across all treatments. The significance of upregulation revealed the important role of CC and CXC chemokine in response to *S. agalactiae* infection in these organs of flowerhorn fish. A significantly higher expression of IL-1*β* was only observed in the liver in all treatments, suggesting that the liver plays a crucial role in the regulation of IL-1*β* production during *S. agalactiae* infection. The significance of upregulation revealed the important role of these inflammatory cytokines in response to *S. agalactiae* infection in the liver and spleen of flowerhorn fish. The liver and spleen are the primary source of immune cells in the defense against bacterial infection of teleost fish as well as the flowerhorn fish. The spleen acts as a secondary lymphoid organ involved in filtering blood and coordinating immune responses, while the liver contributes by producing acute-phase proteins and modulating inflammatory cytokines and chemokines by hepatocytes and Kupffer cells [48].

## 5. Conclusions

This study provides insight into the health-promoting properties of low molecular weight DBPH in flowerhorn fish. Diets supplemented with 2% DBPH exhibited the highest growth, antioxidant activity, and humoral immune response enhancement under normal conditions. Additionally, DBPH could promote the abundance of the genera *Cetobacterium* and *Romboutsia*, which could serve as indicators of the overall health and well-being of the fish. Moreover, with a *S. agalactiae* challenge, fish fed with diets supplemented with DBPH exhibited an enhanced ability to modulate inflammatory genes as well as antioxidant gene expression (SOD and CAT). Overall, dietary supplementation with DBPH could improve the overall health of the flowerhorn fish by ameliorating humoral immune response, alleviating oxidative stress, and strengthening resistance against *S. agalactiae*.

## Figures and Tables

**Table 1:** Primers and target gene sequences for qRT-PCR analysis.

Primer name	5'–3' Nucleotide Sequences	Sequence data	Product size (bp)
SODF	GGAGACCTGGGAAATGTGAC	**GGAGACCTGGGAAATGTGAC**TGCAGGAGCAGATAATGTTGCCAAGATAGACATCACTGACAGTGTGATCAAGCTCACAGGTGCCGACTCCATCATTGGAAGAACCATGGTGATCCATGAGAAGGCTGATGACCTGGGTAAAGGAGGAGATG**AAGAGAGCCTGAAGACTGGT**	171
SODR	ACCAGTCTTCAGGCTCTCTT		

CATF	TCTCAACAGGAACCCAGTCA	**TCTCAACAGGAACCCAGTCA**ATTACTTTGCAGAGGTGGAGCAGCTGGCCTTCGACCCCAGCAACATGCCACCGGGCATTGAGCCCAGCCCTGACAAGATGCTGCAGGGTCGACTCTTCTCCTACCCAGACACACATCGTCACCGGCTTGGGGCA**AACTACCTGCAGATCCCTGT**	174
CATR	ACAGGGATCTGCAGGTAGTT		

IL-1*β*F	GTGACCACTGGCAGAAAGAT	**GTGACCACTGGCAGAAAGAT**CTCGTCCTGTCAGGAGACTTACAGCTGCAGGCCATCACTCTGAAAGGAGGAAACTACCAACACAAAGTGAATTTTAAAATGTCGCGGTACAACTCTTCCTCCGTCACTCCTGGTGAT**GGTCTGACTGTTGTCCTGTC**	150
IL-1*β*R	GACAGGACAACAGTCAGACC		

IL-6F	CCAACCAGCAGTGAGGAG	**CCAACCAGCAGTGAGGAG**CAGATGCTCAAGGTCAACAGTCCGTATGCTTTCCATAGAAAGATGAAAGCGCACAACATCCTGAATCATCTCTTTGATTTCCTCAAAGAAGTGAAAAGATCTATCTGTAGAATGGAGATGAAG**ACCAGGAGAAATATGGCAGC**	191
IL-6R	GCTGCCATATTTCTCCTGGT		

CCF	ACAGAGCCGATCTTGGGTTACTTG	**ACAGAGCCGATCTTGGGTTACTTG**GTCCAGAGAGCAAGGCGTCCATGTGTCAATGCAGTCATCTTTCAGACACAGTCCGGTCTTTTCTGCATCAATGGGAGAGCTCCCTGGGTTCGTGCCACGATTGTTGCATTCGAGAAAGCTAAAGCCCAGTCCACTACACCATCTGTGGTCACTACATCTCCAGTCTCCCTTCTCTCCATC**ATAACATCCACCGCCTCTCCTTCA**	229
CCR	TGAAGGAGAGGCGGTGGATGTTAT		

CXCF	TGTCTGTGTCACCGTGTCAGGAAT	**TGTCTGTGTCACCGTGTCAGGAAT**CGTGTTGGCCTGAAGTCGGAAATAAAGGACATTCAGATCTACCCAGCAACCATCTTCTGCAACAAAGTGGAGATTGTTGTCACCTTGAACAACAGCTATCGCT**ATTGCTTGAACCCTGAGCTGAAGG**	151
CXCR	CCTTCAGCTCAGGGTTCAAGCAAT		

*β*-actinF	ACAGGATGCAGAAGGAGATCACAG	**ACAGGATGCAGAAGGAGATCACAG**CCCTGGCCCCATCCACCATGAAGATCAAGATCATTGCCCCACCTGAGCGTAAATACTCCGTCTGGATCGGAGGCTCCATCCTGGCCTCCCTGTCCACCTTCCAGCAG**ATGTGGATCAGCAAGCAGGAGTAC**	155
*β*-actinR	GTACTCCTGCTTGCTGATCCACAT		

*Note:* Bases in bold indicate the position and sequence of the primers.

**Table 2:** Immune parameters of flowerhorn fish fed experimental diets for 30 days.

Paramaters	Treatments						
	Control	0.1% Vitamin C	0.5% DBPH	1% DBPH	2% DBPH	RSE	*p*-Value
ACH50 (units/mL)	23.67^a^	29.28^b^	25.27^a^	25.51^a^	30.18^b^	2.52	<0.001
Lysozyme activity (µg/mL)	15.41^a^	16.63^b^	16.80^b^	16.31^b^	16.51^b^	0.51	0.001
Total Ig (mg/mL)	1.29^a^	1.51^b^	1.33^a^	1.51^b^	1.64^b^	0.10	<0.001

*Note*: Means with different superscripts in each row differ significantly (*p* < 0.05).

Abbreviations: ACH50, alternative complement activity; DBPH, duck blood protein hydrolysate; RSE, residual standard error; Total Ig, total immunoglobulin.

**Table 3:** Antioxidant parameters of flowerhorn fish fed experimental diets for 30 days.

Parameters	Treatments						
	Control	0.1% Vitamin C	0.5% DBPH	1% DBPH	2% DBPH	RSE	*p*-Value
SOD (U/mL)	2.63^a^	4.61^b^	3.74^ab^	3.82^b^	4.12^b^	0.47	0.003
MDA (nmol/mL)	4.14^b^	1.15^a^	4.39^b^	2.18^a^	1.24^a^	1.40	<0.001
CAT (nmol/min/mL)	17.74^a^	23.81^b^	23.59^b^	24.50^b^	23.99^b^	2.49	0.013

*Note:* Means with different superscripts in each row differ significantly (*p* < 0.05).

Abbreviations: CAT, catalase; DBPH, duck blood protein hydrolysate; MDA, malondialdehyde; RSE, residual standard error; SOD, superoxide dismutase.

**Table 4:** Effects of flowerhorn fish fed 2% DBPH for 30 days on the diversity of the microbiome in the intestinal tract.

Parameter		Treatments						
		Control	2% DBPH			RSE		*p*-Value
Raw read		80.137	80.009			189.63		0.458
Clean read		72.201	72.084			580.80		0.817
Tags		71.203	71.558			645.59		0.538
OTU		562.00^a^	316.67^b^			47.34		0.010
Chao1		563.95^a^	318.20^b^			47.34		0.010
Shannon		4.15^a^	3.14^b^			0.26		0.009
Simpson		0.82	0.74			0.04		0.143

*Note:* Means in the same row sharing different superscripts were significantly different as determined by an independent-sample *t*-test at the significance level accepted at *p* < 0.05.

Abbreviations: DBPH, duck blood protein hydrolysate; RSE, residual standard error.

## Data Availability

The original contributions presented in the study are included in the article and further inquiries can be directed at the corresponding author.
